# *Alcyonidium kuklinskii* sp. nov., a new species of Antarctic ctenostome bryozoan with a key to all Antarctic species of the genus

**DOI:** 10.1007/s13127-023-00629-4

**Published:** 2023-12-08

**Authors:** Thomas Schwaha, Valentina Cometti, Ahmed J. Saadi, Matteo Cecchetto, Stefano Schiaparelli

**Affiliations:** 1 Department of Evolutionary Biology, University of Vienna, Schlachthausgasse 43, 1030 Vienna, Austria; 2 Italian National Antarctic Museum (MNA), Section of Genoa, Genoa, Italy; 3 Department of Earth, Environmental and Life Science (DISTAV), University of Genoa, Genoa, Italy

**Keywords:** Alcyonidioidea, Polar bryozoans, Identification key, Distribution

## Abstract

Recent surveys of Antarctic waters in the Terra Nova Bay (Ross Sea) revealed numerous bryozoan species including ctenostome bryozoans. Whereas cheilostome bryozoans are well-studied in these latitudes, ctenostomes remain highly neglected. Large ctenostomes are easily recognized by their lack of calcified skeletons, but this lack also renders them difficult and tedious to identify. As a result, histology and reconstructions of internal soft tissues are required to classify this group of bryozoans. Thanks to the availability of new specimens from Terra Nova Bay, a detailed analysis of growth form, gut morphology and tentacle number of two colonies, initially ascribed to the ctenostome bryozoan genus *Alcyonidum* Lamouroux, 1813, turned out to be a new species, *Alcyonidium kuklinskii* sp. nov., which we described in this study. These specimens were also barcoded (COI) and sequences compared to available ones. Together with the new species described here, a total of ten species of *Alcyonidium* is now known for the Southern Ocean, accounting for one eighth of the entire genus diversity. All Southern Ocean species appear to be endemic. In order to speed the identification of the Antarctic *Alcyonidium* species, we provide an identification key and a distribution map of all type species. In brief, colony morphology, zooidal size and, in particular tentacle number represent the most suitable characters for identifying species within this genus.

**Supplementary Information:**

The online version contains supplementary material available at 10.1007/s13127-023-00629-4.

## Introduction

Bryozoans are a phylum of colonial suspension feeders belonging to Lophotrochozoa among Protostomia (Bleidorn et al. 2019). Colonies are formed by asexual buds resulting in clonal individuals, termed zooids, that comprises a tentacle crown or lophophore, u-shaped gut and diverse, associated neuromuscular tissue that is generally referred to as ‘polypide’. The protective body-wall, the cystid (Mukai et al., 1997; Schwaha et al., 2020), can be mineralized in the clades Stenolaemata and Cheilostomata, which represent the most speciose and dominant taxa among bryozoans (Taylor, 2020).

Ctenostome bryozoans represent a small group of unmineralized bryozoans with approximately 350 recent species (Schwaha 2020). They show a multitude of different colony and zooid morphologies ranging from erect, encrusting to even boring/endolithic forms; and box-shaped to highly elongated, tubular zooids. One of the largest, more easily encountered and addressed ctenostome genera is *Alcyonidium* Lamoroux, 1813. It forms rather large colonies that can be erect or encrusting. Species are often transparent, but in several cases yellowish to brown coloration are found (D’Hondt 1983). Species identification is a long-lasting problem in the genus and superficial observations led to many misidentifications in the past (see e.g. Cadman & Ryland, 1996; Porter et al., 2001; Ryland & Porter, 2003; Porter, 2004). While molecular sequencing has made some advances in the past decades (see also references above), morphological characters still remain the most widely used tool for identification and involves typical characters such as colony and zooidal morphology, presence of kenozooids, but also features such as tentacle number, reproductive mode (Porter & Hayward, 2004) and even details of gut morphology (Le Brozec, 1955, D’Hondt, 1983). The latter usually requires either fine dissections or histological analyses.

A recent survey on the bryozoan species of Terra Nova Bay in the Ross Sea, housed in the collection of the Italian National Antarctic Museum, revealed 127 different species from 75 different sampling sites. Most species belong to cheilostome bryozoans (80%) and cyclostomes (18%) whereas ctenostomes account for just 2% of the entire collection (Cecchetto et al., 2019). In this first survey, a species of *Alcyonidium* remained unidentified (Fig. 1 in Cecchetto et al., 2019), and preliminary analysis by the authors of this study revealed it to represent a yet undescribed species.

**Fig. 1:**
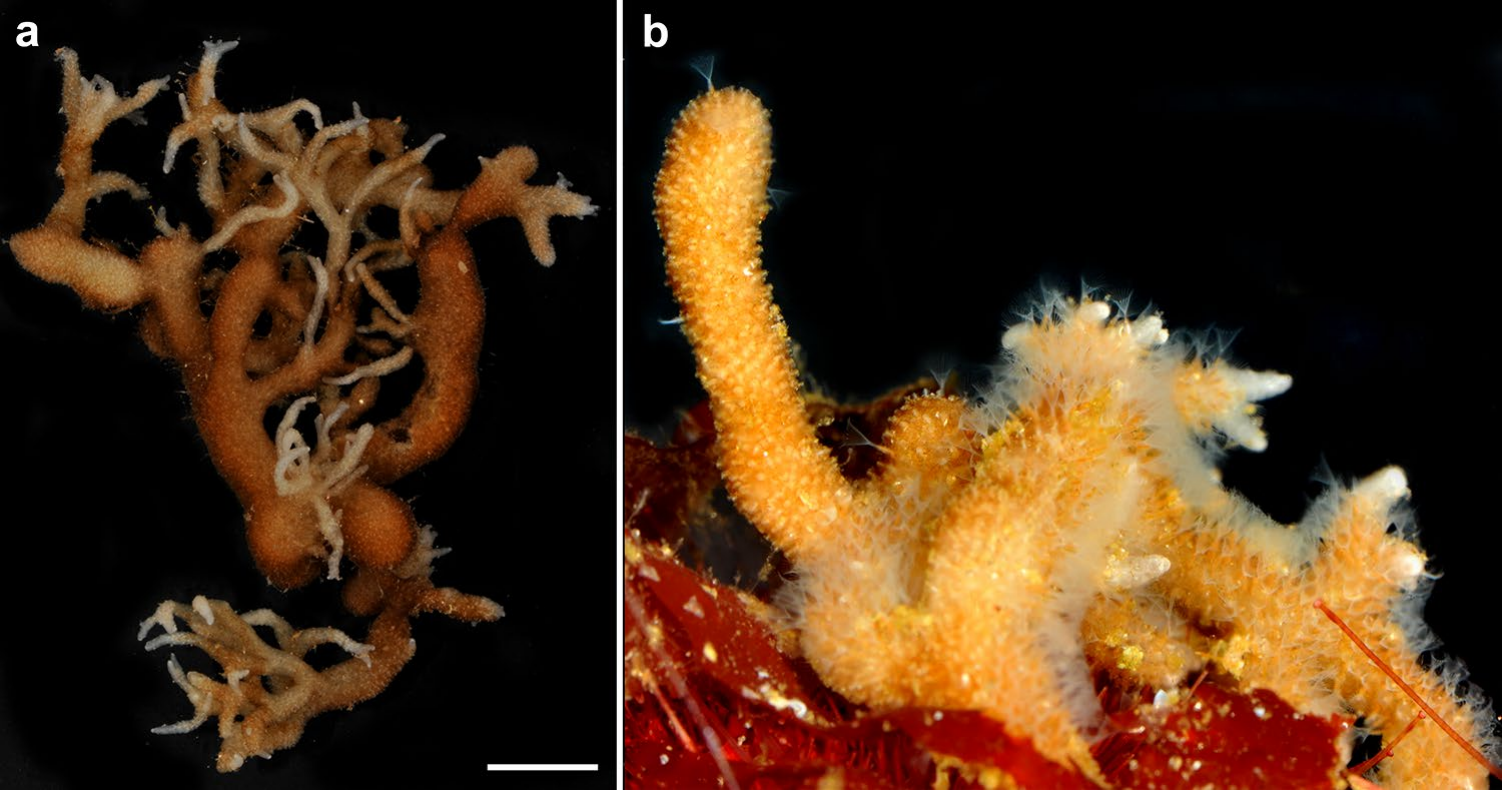
Live specimens of *Alcyonidium kuklinskii* sp. nov., modified from Cecchetto et al. (2019). **a** Holotype MNA-02733 Overview of a large colony showing thick and thinner erect branching (scale bar = 1 cm). **b** Paratype, MNA-02904. Detail of few branches with partially extended lophophores

In this study, we provide a formal description and a detailed analysis of the new species, *Alcyonidium kuklinskii* sp. nov., together with an identification key and a short biogeographical assessment of the species occurring in the Southern Ocean. Thereby, we hope to provide a helpful tool in species identification in all future studies of Antarctic *Alcyonidium* and to give a better tool for biodiversity assessment of Antarctic faunas.

## Material and methods

### Material

Two specimens of *Alcyonidium* sp. were collected in the framework of the XXV expedition of the Italian National Antarctic Program (PNRA) by one of the authors (SS) in Terra Nova Bay of Ross Sea during two separate SCUBA dives at the same location, named “Zecca” (74°41′24.972″ S, 164°6′9.18″ E), at a depth of 26 m. This site is already the type locality of another species, the calcareous sponge *Megapogon schiaparellii* Alvizu et al., 2019 (Alvizu et al., 2019). One portion of a specimen (MNA voucher code MNA-02733, field code XXV-20, collected on 10 December 2009) was stored in paraformaldehyde, another in absolute ethanol and the rest kept at − 20 °C, whereas the other specimen (voucher code MNA-02904, field code XXV-91, collected on 12 December 2009) was stored in absolute ethanol. Both specimens are now stored at the Italian National Antarctic Museum (MNA, section of Genoa).

Samples of *Alcyonidium gelatinosum* were collected by dredging of dead bivalve shells at the Bay of Morlaix, Chateau du Taureau (France) in 2021. Samples were fixed in 96% ethanol.

### Methods

#### Morphological methods

Documentation of colony pieces was conducted with a Nikon SMZ25 (Nikon, Tokyo, Japan) equipped with a Nikon DsRi-2 microscope camera, or a Hirox RH2000 microscope. For histological analysis, short branches of the colony were cut off, dehydrated in a graded ethanol series and afterwards infiltrated and embedded into Agar Low Viscosity Resin (Agar Scientific, Stansted, UK). Serial sections were conducted according to Ruthensteiner (2008). Sections were stained with toluidine blue, documented and analysed with a Nikon NiU microscope.

#### Molecular methods

##### DNA extraction and sequencing

One portion of specimen MNA-02904 was clipped from the colony and transferred to a “plant tube rack” following the instructions provided by the Canadian Centre for DNA Barcoding (CCDB, https://ccdb.ca/site/wp-content/uploads/2019/07/Instructions_Plant.pdf). The DNA extraction was performed according to the CTAB protocol (https://ccdb.ca/site/wp-content/uploads/2016/09/CCDB_DNA_Extraction-Plants.pdf). Amplification and sequencing were carried out at the CCDB, targeting the partial cytochrome c oxidase subunit 1 (COI-5P) with the primer pair LCO1490_t1 and HCO2198_t1 (forward and reverse respectively, Foottit et al. 2009).

For *Alcyonidium gelatinosum* DNA was extracted using the QIAamp DNA Micro Kit (QIAGEN, Hilden, Germany) following the manufacture’s protocol. Approximately 650 nucleotides of the COI gene were PCR amplified using primers LCO 1490 and HCO 2198 (Folmer et al., 1994). The PCR amplification of COI gene was performed using Red HS Taq Master Mix (Biozym, Oldendorf, Germany) (30 µl reaction with 1 µm of 20 µM primer, 1–3 µl of extracted DNA and 15 µl of Red HS Taq Master Mix). PCR products were cleaned using an enzymatic clean up reagent A’SAP (ArcticZymes Technologies ASA, Tromsø, Norway) and send to Microsynth Austria GmbH for sequencing.

Five *Alcyonidium* species were also included from publicly available data. The following samples were retrieved from GenBank (*A. mamillatum*: FJ196100 (Fuchs et al., 2009), *Alcyonidium* sp.: OQ32326 and *A. verrilli*: OQ323354. The COI sequence for *A. flabelliforme* was obtained from the whole genome assembly of *A. flabelliforme* available on GenBank under accession number: JAOQFJ010000003 using Exonerate v2.4.0 (Slater & Birney, 2005) with affine:local model and then annotated using MITOS2 web server (Donath et al., 2019). For *A. polyoum*, COI sequence was obtained from the transcriptome assembly of *A. polyoum* available on Dryad repository (https://doi.org/10.5061/dryad.95X69p8n1) (Saadi et al., 2022) as described above.

##### Phylogenetic analysis

The COI sequences were aligned using MAFFT 7.310 (Katoh et al., 2002) with the following options: –auto, –localpair and –maxiterate 1000. The alignment was then trimmed manually in order to remove ambiguously aligned sites. Maximum likelihood (ML) analysis was performed with IQ-TREE2 v2.1.2 (Minh et al., 2020) using ModelFinder tree search with 1000 ultrafast bootstraps and SH-aLRT test replicates (Hoang et al., 2018; Kalyaanamoorthy et al., 2017). Bayesian analysis (BI) was performed using the MrBayes (version 3.2.7a) package (Ronquist et al., 2012) with two separate runs of four chains of a Markov Chain Monte Carlo (MCMC) algorithm. BI analysis was conducted for two million generations with tree sampling every 100 generations. The run ended only after the Bayesian MCMC searches had reached a stationary phase (indicating convergence of the chains onto the target distribution) . A consensus tree was calculated using the last 75% best scoring trees and 25% of the sampled trees were discarded as burn-in. Finally, ML-corrected substitutions per site were calculated in MEGA 7 using the maximum composite likelihood parameter with a gamma parameter of 1.0 (Kumar et al., 2008).

## Results

### Description of new Antarctic species of Alcyonidium

**Gymnolaemata**

*Ctenostomata* (paraphyletic)

**Alcyonidioidea**

**Genus** *Alcyonidium.*

*Alcyonidium kuklinskii* sp. nov.

*Alcyonidium* sp. Cecchetto et al., 2019, Fig. 1

**Etymology**: Named after bryozoologist Piotr Kuklinski for his contribution to polar bryozoan research.

**Type locality**: Ross Sea, Terra Nova Bay, “Zecca” diving site, Latitude: 74°41′24.972″ S, Longitude: 164°6′9.18″ E.

**Types**: Holotype: MNA-02733, Paratype: MNA-02904.

**Fig. 2:**
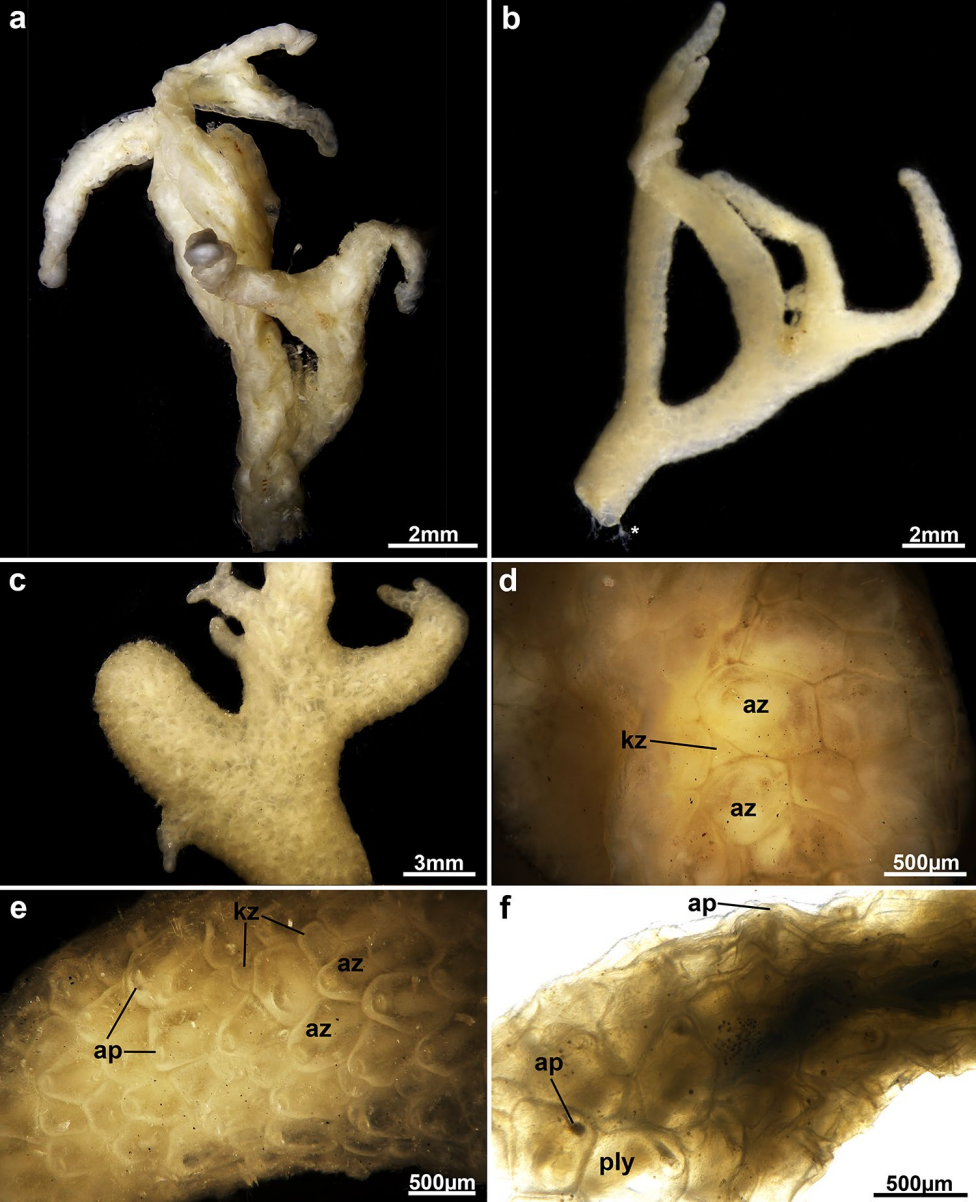
Stereomicroscopic images of *Alcyonidium kuklinskii* sp. nov. Images **a**–**c**, **e**–**f** from holotype MNA-02733, **d** from paratype MNA-02904, **a** and **b** Colony pieces of the holotype, **c** Close-up showing thinner and thicker branches of the colony. **d** and **e** Zooidal shapes and few small kenozooids in between. **f** More cleared colony piece showing internal, functional polypides. Abbre: ap – aperture, az – autozooid, kz – kenozooid, ply - polypide

**Fig. 3:**
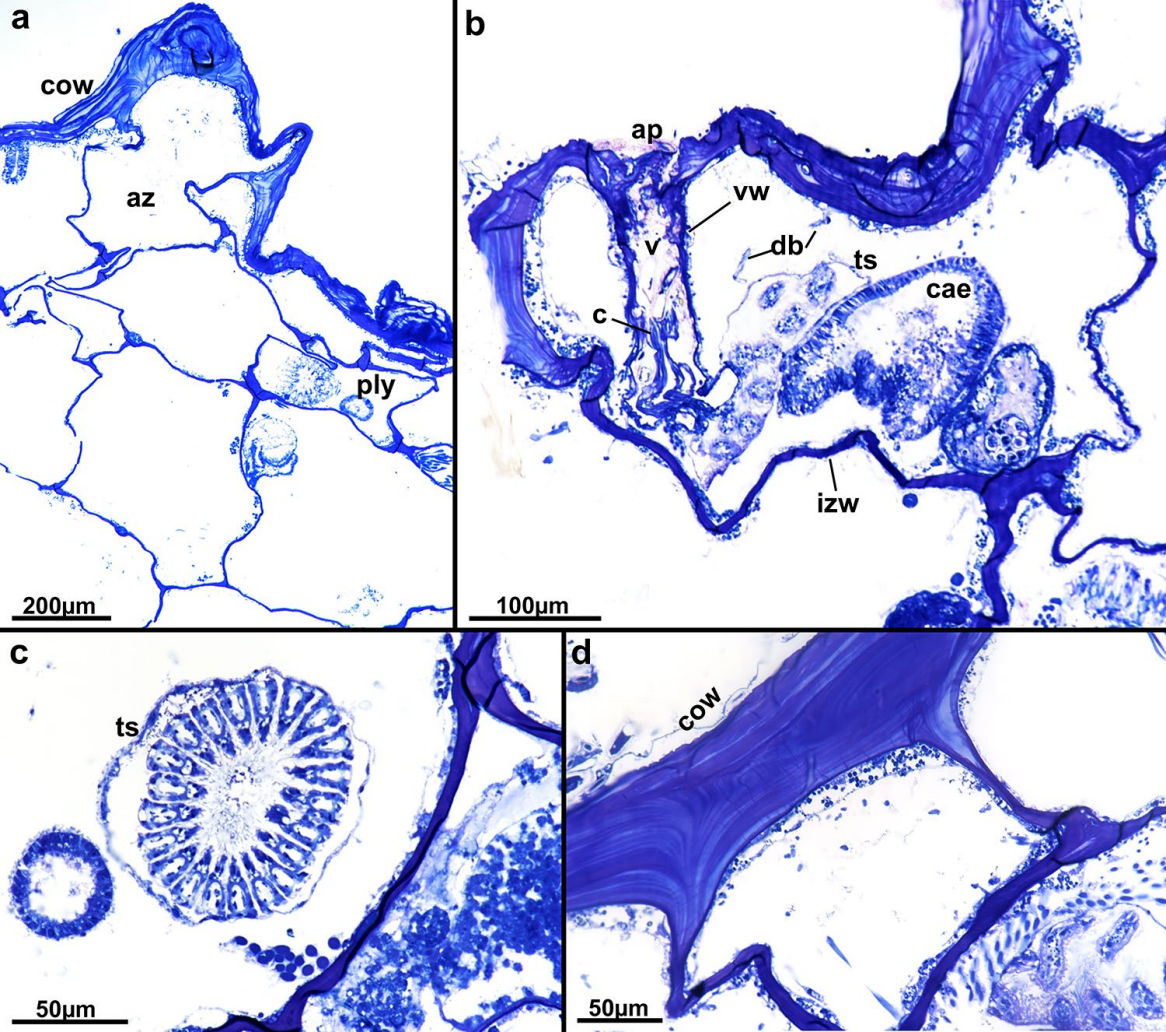
Histological sections of *Alcyonidium kuklinskii* sp. nov. **a** Multiple internal zooids filling the internal branch. Note only close to the colony wall there are functional polypides. **b** Apertural area showing deep vestibular wall and medium-sized collar at its bottom. Parts of the remaining polypide are also visible. **c** Cross-sectioned lophophore showing 21 tentacles. **d** Detail of the thick, multi-layered colony wall. Abbre: az – autozooid, c – collar, cae – caecum, cow – colony wall, db – duplicature band, izw – interzooidal wall, ply – polypide, ts – tentacle sheath, v – vestibulum, vw – vestibular wall

**Fig. 4:**
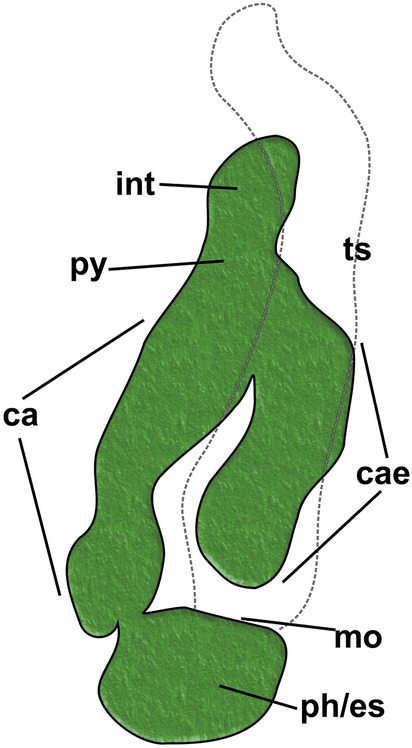
Schematic drawing of the digestive tract anatomy of *Alcyonidium kuklinskii* sp. nov. Abbre: ca – cardia, cae – caecum, int – intestine, mo – mouth opening, ph/es – pharynx/esophagus, py – pylorus, ts – tentacle sheath

**Diagnosis**: Colony erect and non-pedunculate, cylindrically branching forming thick main branches and sometimes thin smaller ones (Figs. 1, 2a–c, see also Fig. 1 in Cecchetto et al., 2019). Thick branches consisting of internal medullary, old zooids (Fig. 3a). Zooids hexagonal to polygonal, measuring between 490 and 610 µm in length and 370–530 µm width (Fig. 2d–f). Small kenozooids of triangular or polygonal shape irregularly present on frontal surface (Fig. 2d, e). Apertural papilla missing, vestibular wall extends to basal side of zooid (Fig. 3b). External cuticle thick and multi-layered (Fig. 3d). Lophophore with 21–22 tentacles (Fig. 3c), digestive tract with short foregut, very elongated cardia, prominent elongated caecum folding into proximal direction almost until cardiac valve, anus vestibular (Fig. 4). Reproduction unknown.

**Remarks**: From all Antarctic alcyonidiid species four are encrusting (*A. antarcticum* Waters, 1904, *A. eightsii* Winston & Hayward, 1994, *A. epispiculum* Porter & Hayward, 2004, *A. simulatum* Porter & Hayward, 2004), five erect (*A. austral*e D’Hondt & Moyano, 1979, *A. flabelliforme* Kirkpatrick, 1902, *A. kuklinskii* sp. nov., *A. scolecoideum* Porter & Hayward, 2004, *A. torpedo* D’Hondt, 2006) and one pelagic (*A. pelagosphaerum* Porter & Hayward, 2004), *A. kuklinskii* is the only erect, cylindrical form with 21–22 tentacles. *Alcyonidium australe* is colony-wise the most similar species but easily identifiable by its lower number of tentacles (16–17). Tentacle-wise only *A. eightsii* and *A. epispiculum* have similar numbers (20–21 and 22–23, respectively), but differ in being encrusting species (see Table 1). In addition, zooids are much longer in *A. eightsi* (0.8–1 mm) contrary to *A. kuklinskii*, 0.49–0.61 mm. The length of *A. epispiculum*, 0.43–0.54 mm, is more similar to *A. kuklinskii* but are much broader, 0.51–0.71 mm, compared to 0.37–0.53 mm. Details of gut morphology have been previously used for alcyonidiid species discrimination (Le Brozec, 1955), but remain unstudied in all other Antarctic species.

**Table 1:** Comparison of all Antarctic species of *Alcyonidium*, modified and extended from Porter et al. 2004

**Species**	**Growth form**	**Zooid width**	**Zooid length**	**Tentacle number**	**Reproductive mode**
*A. antarcticum* Waters, 1904	Encrusting (echinoderm spines)	?	> 1 mm	24–27	Planktotrophic
*A. australe* d'Hondt & Moyano, 1979	Erect, thick, branching, cylindrical			16–17	Planktotrophic
*A. eightsi* Winston & Hayward, 1994	Encrusting, unilaminar sheet	0.46–0.56 mm	0.8–1 mm	22–23	Lecithotrophic
*A. epispiculum* Porter & Hayward, 2004	Encrusting (echinoderm spines)	0.51–0.71 mm	0.43–0.54 mm	20–21	Planktotrophic
*A. flabelliforme* Kirkpatrick, 1902	Erect, foliate, pedunculate	0.51–0.61 mm	0.59–0.69 mm	27–31	Planktotrophic
*A. kuklinskii* sp. nov	Erect, thick, chunky	0.37–0.53 mm	0.49–0.61 mm	21–22	?
*A. pelagosphaerum* Porter & Hayward, 2004	Spherical, pelagic				
*A. scolecoideum* Porter & Hayward, 2004	Erect, long & slender			28–29	Planktotrophic
*A. simulatum* Porter & Hayward, 2004	Irregular sheet, encrusting	0.46–0.58 mm	0.93–1.27 mm	29–30	Planktotrophic
*A. torpedo* D’Hondt, 2006	Erect, pedunculate, foliate, ‘pancake’-like		1.6 mm		?

### Barcode sequence

The specimen analysed, MNA-02904, was sequenced to obtain a final COI sequence length of 646 bp. Taxonomic assignation was checked through Barcode of Life database (BOLD) and National Center for Biotechnology Information (NCBI) database BLAST (https://blast.ncbi.nlm.nih.gov/Blast.cgi, accessed on 09 June 2023) for definitive assignment. A sequence match > 98% with the reference database was considered an 'exact' match (Leray & Knowlton, 2016). The other Antarctic species with available sequences, *Alcyonidium flabelliforme*, did not match our reported species *A. kuklinskii* sp. nov.. The sequence of *A. kuklinskii* sp. nov is available at the Barcode of Life Data System (BOLD, Ratnasingham and Hebert, 2007) with the processID BAMBI2007-20. Genbank accession number is OR076125. Sequence data of *A. gelatinosum* is accessible under OR187863 Genbank accession number.

### Molecular phylogeny

Sequences of the COI gene were generated for two *Alcyonidium* species and combined with five publicly available COI sequences of *Alcyonidium*. ML phylogenetic tree was constructed using of 646 unambiguously aligned nucleotide sites of COI gene. *Pectinatella magnifica*: NC_038192 (Gim et al., 2018) was used as an outgroup to root the phylogenetic tree.

**Fig. 5:**
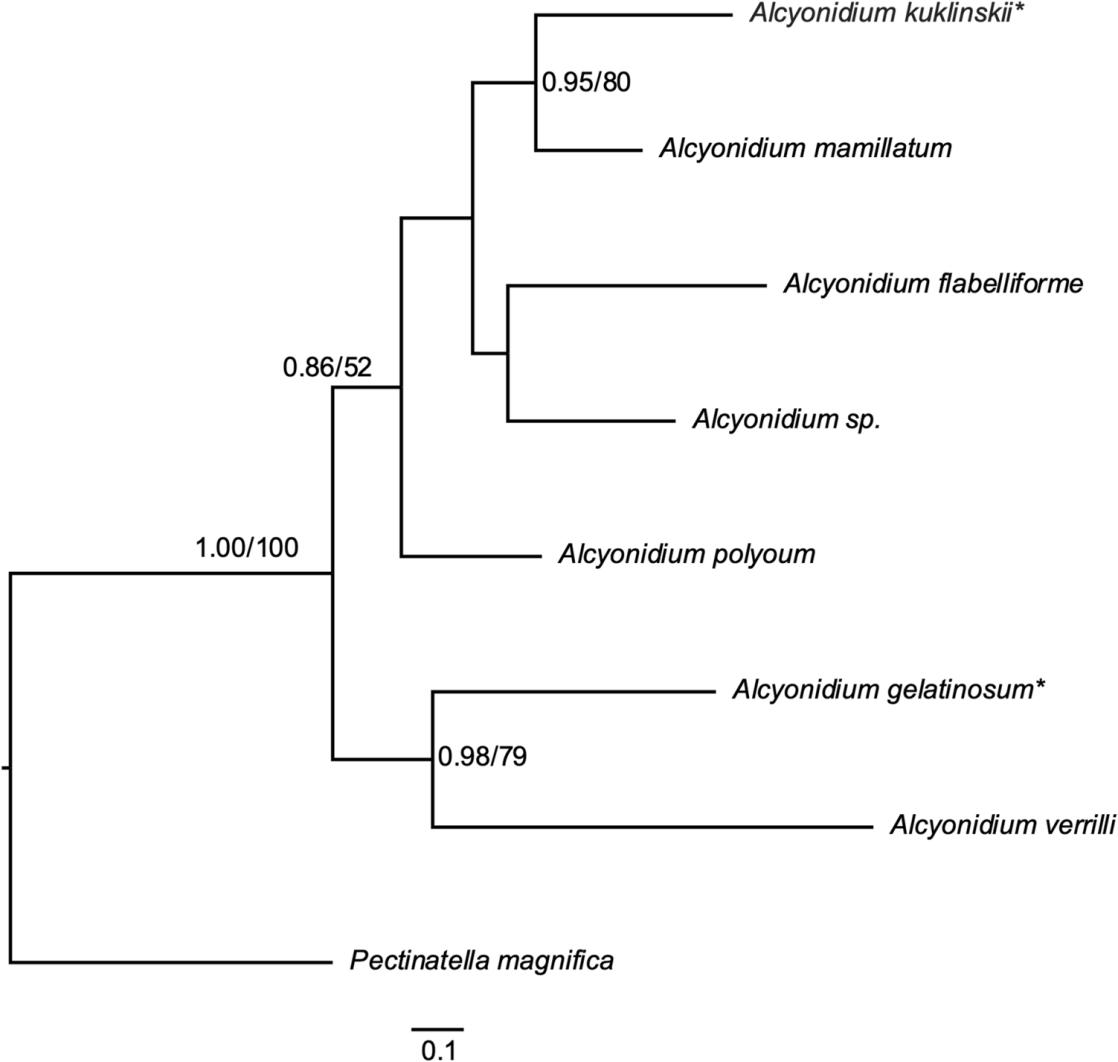
Maximum likelihood phylogenetic tree of Alcyonidium based on 646 unambiguously aligned nucleotide sites of the COI gene. Values on nodes represent posterior probabilities for BI (based on last 75% of trees) and bootstrap support (1000 replicates), respectively. Support values < 50% are not shown. The scale bar represents one substitutional change per 100 nucleotide positions. * Sequence was generated during this study

The ML and BI phylogenies resulted in highly consistent topologies with little variation in support values as shown in Fig. 5 in the ML topology and in BI tree in the supplementary information Fig. S1. Two clades were recovered in the *Alcyonidium* phylogeny, the first clade comprising *A. gelatinosum* and *A. verrilli* supported with 0.98 Bayesian posterior probabilities (PP) and 79 bootstrap replicates. The second clade includes the remaining *Alcyonidium* species and is supported with 0.86 PP and 52 bootstraps. Within the latter clade, the new *Alcyonidium* species and *A. mamillatum* have a sister taxon relationship supported in 0.95 PP and 80 bootstraps.

The pairwise ML-corrected distances over the COI gene sequences between all *Alcyonidium* species are provided in Table 2. Pairwise ML distances ranged from 0.300 between *Alcyonidium kuklinskii* sp. nov. and *A. mamillatum* to 0.565 between *A*. *gelatinosum* and *Alcyonidium kuklinskii* sp. nov..

**Table 2:** Pairwise ML-corrected genetic distances observed in the mitochondrial cytochrome c oxidase subunit I (COI) sequences between all *Alcyonidium* species

Species	1	2	3	4	5	6	7
1-* A. flabelliforme*	-						
2-* A. polyoum*	0.381	-					
3- *A. kuklinskii* sp. nov	**0.383**	**0.367**	-				
4-* A. mamillatum*	0.379	0.310	**0.300**	-			
5- *Alcyonidium* sp.	0.382	0.368	**0.348**	0.311	-		
6-* A. gelatinosum*	0.504	0.409	**0.565**	0.442	0.376	-	
7*- A. verrilli*	0.506	0.510	**0.542**	0.524	0.476	0.337	-

**Table:** 

**Key to the Antarctic species of *Alcyonidium***	
1. Colony pelagic	*A. pelagosphaerum*
Colony benthic	2
2. Colony encrusting	3
Colony erect	6
3. Encrusting mostly erect, frondose bryozoans or algae	4
Encrusting sea urchin spines as a single layer	5
4. Colony brooding embryos, 22-23 tentacles	*A. eightsi*
Colony not-brooding embryos, 29-30 tentacles	*A. simulatum*
5. Zooids with slightly to enlarged apertures, 24-27 tentacles	*A. antarcticum*
Zooids with no apparent apertural papillae, small kenozooids present on frontal surface, 20-12 tentacles	*A. epispiculum*
6. Erect growth form cylindrical	7
Erect growth form flattened and pedunculate	8
7. Zooids with 16-17 tentacles	*A. australe*
Zooids with 21-22 tentacles	*A. kuklinskii*
Zooids with indistinct zooidal boundaries, 28-29 tentacles	*A*. *scolecoideum*
8. Colony flat forming single large flab with zooids shorter than 1mm	*A. flabelliforme*
Colony pancake-shaped with zooid extending 1mm	*A. torpedo*

### Geographical distribution of Antarctic alcyonidiids

**Fig. 6:**
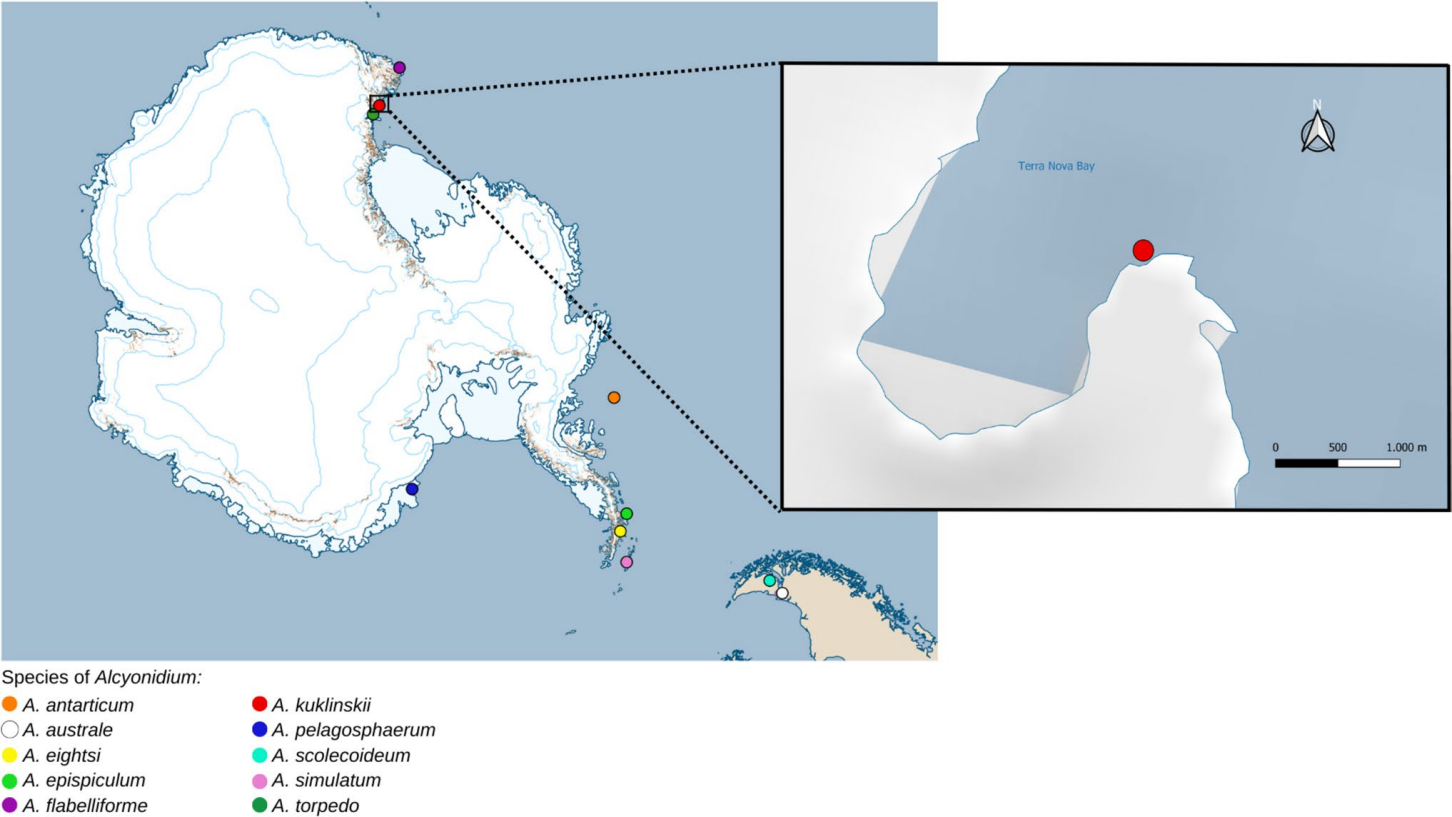
Map showing the type localities of all Antarctic species of *Alcyonidium*

Five species of Antarctic *Alcyondium* are recorded from the Straight of Magellan and the Antarctic Peninsula (see Table 3): *A. australe*, *A. eightsi*, *A. epispiculum*, *A. scolecideum*, *A. simulatum*. *A. antarcticum* is found close to the peninsula in the Bellinghausen Sea. The pelagic and still almost unknown *A. pelagosphaerum* occurs in the Weddell Sea. Types of *A. flabelliforme* and *A. kuklinskii* sp. nov. have been found in the Ross Sea, with *A. torpedo* having a close collection site (Fig. 6).

**Table 3:** Geographical distribution of Antarctic type species of *Alcyonidium*

**Species**	Locality	Depth
*A. antarcticum* Waters, 1904	Bellingshausen Sea	459 m
*A. australe* d'Hondt & Moyano, 1979	Straight of Magellan	shallow?, 40–49 m, up to 310–360 m
*A. eightsi* Winston & Hayward, 1994	Low Island, South Shetland Islands, Palmer Archipelago	50–70 m
*A. epispiculum* Porter & Hayward, 2004	Palmer Archipelago	70–150 m
*A. flabelliforme* Kirkpatrick, 1902	Cape Adare, Robertsons bay	82–92 m
*A. kuklinskii* sp. nov	Terra Nova Bay	26 m
*A. pelagosphaerum* Porter & Hayward, 2004	Halley Bay, Weddel Sea	no data
*A. scolecoideum* Porter & Hayward, 2004	Inutil Bay, Straight of Magellan	22–26 m
*A. simulatum* Porter & Hayward, 2004	Nelson Island, South Shetland Islands	64–82 m
*A. torpedo* D’Hondt 2006	Adélie land	100 m

## Discussion

### Diversity of polar species of *Alcyonidium*

As previously already indicated (Porter & Hayward, 2004), there seems to be a high degree of endemism among Antarctic species of *Alcyonidium*. So far, all ten species seem to be restricted to the Antarctic and Subantarctic waters, with some occurring in the southernmost tip of South America. With currently ~ 80 species described in the genus, the Southern alcyonidiids would entail one eight of all known species. Although some older species descriptions are very limited and provide little details (e.g. Waters, 1904, D’Hondt 2006), we were able to provide a key to all known Antarctic species of *Alcyonidium* based on all available information that should allow for easier species discrimination in the future.

Up to 15 species or variants have been reported from Arctic waters (Kluge, 1975), but many were assigned to species known from more temperate, particularly European waters such as *Alcyonidium gelatinosum*, *A. mytili*, or *A. diaphanum*, which were particularly prone to being misidentified in the past (Cadman & Ryland, 1996; Porter et al., 2001, Ryland & Porter 2003). Unfortunately, no detailed analyses employing morphological and basic molecular techniques have been applied to Arctic species yet. This would ultimately clarify the true identity of these species and also show whether a similar degree of endemism is present in Arctic waters. One particularly unique species, however, truly endemic to Arctic areas is *A. disciforme*, which forms unattached, disc-shaped colonies (Kuklinski & Porter, 2004).

### Distribution of Antarctic alcyonidiids

As evident from the localities from all described species, most species have been described from the Straight of Magellan, or locations around the Antarctic Peninsula, such as the South Shetlands Islands, whereas other areas show a distribution over dispersed localities. This is most likely a result of sampling bias because of extensive collections from the US Antarctic Research Program (USAP), which were also thoroughly studied (Porter & Hayward, 2004). Besides sampling, taxonomic expertise is another requirement for ctenostome animals, especially since it usually requires histological information for proper species identification and diagnosis. We estimate that the diversity of Antarctic alcyonidiids might be much higher, but requires more study in the future.

### Characters in *Alcyonidium* systematics

As mentioned, *Alcyonidium* represents one of the largest genera of ctenostome bryozoans (www.bryozoa.net) and has traditionally been one of the most difficult to identify and many species have been wrongly addressed or identified in the past (see references above). Particularly, features such as reproduction and tentacle number have proved to be very important for species discrimination. Also, internal features of the digestive tract have long been pointed out as very informative character for species identity (Le Brozec, 1955, see also D’Hondt, 1983). Unfortunately, this information is seldom recorded in more recent species description, but has proven also very useful in closely-related families such as the Pherusellidae (Decker et al., 2021).

Soft-tissue or reproductive characters remain the most useful characters for species determination in all ctenostome bryozoans (Jebram, 1986; Schwaha, 2020). The current assessment on Antarctic bryozoans shows that colony morphology and tentacle number essentially can provide the necessary features for species discrimination. In many preserved material or broken pieces of colonies this will practically be difficult to test without dissection or histology. Also, some broken pieces of colonies might either lack polypides or be insufficient altogether for proper species identification (see also Porter & Hayward, 2004). Polypide features especially the variable gut structure would be important to evaluate in the future, as it could represent an easy and reliable tool for species discrimination by simple mounting of bryozoan guts.

Reproduction is likewise a character of important species assignment, but very difficult to assess in most cases as it often required histology to check: size and number of gonads (oocytes), presence of an intertentacular organ (ITO, used in planktotrophic species), presence of brooded large embryos. Also, in many species the mode of reproduction is not evident when lacking embryos or distinct gonads as encountered in our current analysis on *Alcyonidium kuklinskii* sp. nov..

Ultimately, it will be crucial that more genetic data along with proper morphological identification will be provided for many species of *Alcyonidium*. This would ease the difficulties in species identification and biodiversity assays in the future.

### Supplementary Information

Below is the link to the electronic supplementary material.

Supplementary file1: Bayesian phylogenetic tree of *Alcyonidium* based on 646 unambiguously aligned nucleotide sites of the COI gene. Values on nodes represent posterior probabilities (based on last 75% of trees). Support values < 50% are not shown. The scale bar represents one substitutional change per 100 nucleotide positions. * Sequence was generated during this study (PNG 243 KB)

## Data Availability

Data is available on reasonable request. Trees and alignment can be found: https://figshare.com/s/f9e54b785771fcd233e5
