# Supplementary figures and images for: Alcyonidium kuklinskii sp. nov., a new species of Antarctic ctenostome bryozoan with a key to all Antarctic species of the genus
Source: Org Divers Evol. 2023 Dec 8;24(1):85–94. doi: 10.1007/s13127-023-00629-4 (PMC10927890; doi:10.1007/s13127-023-00629-4)

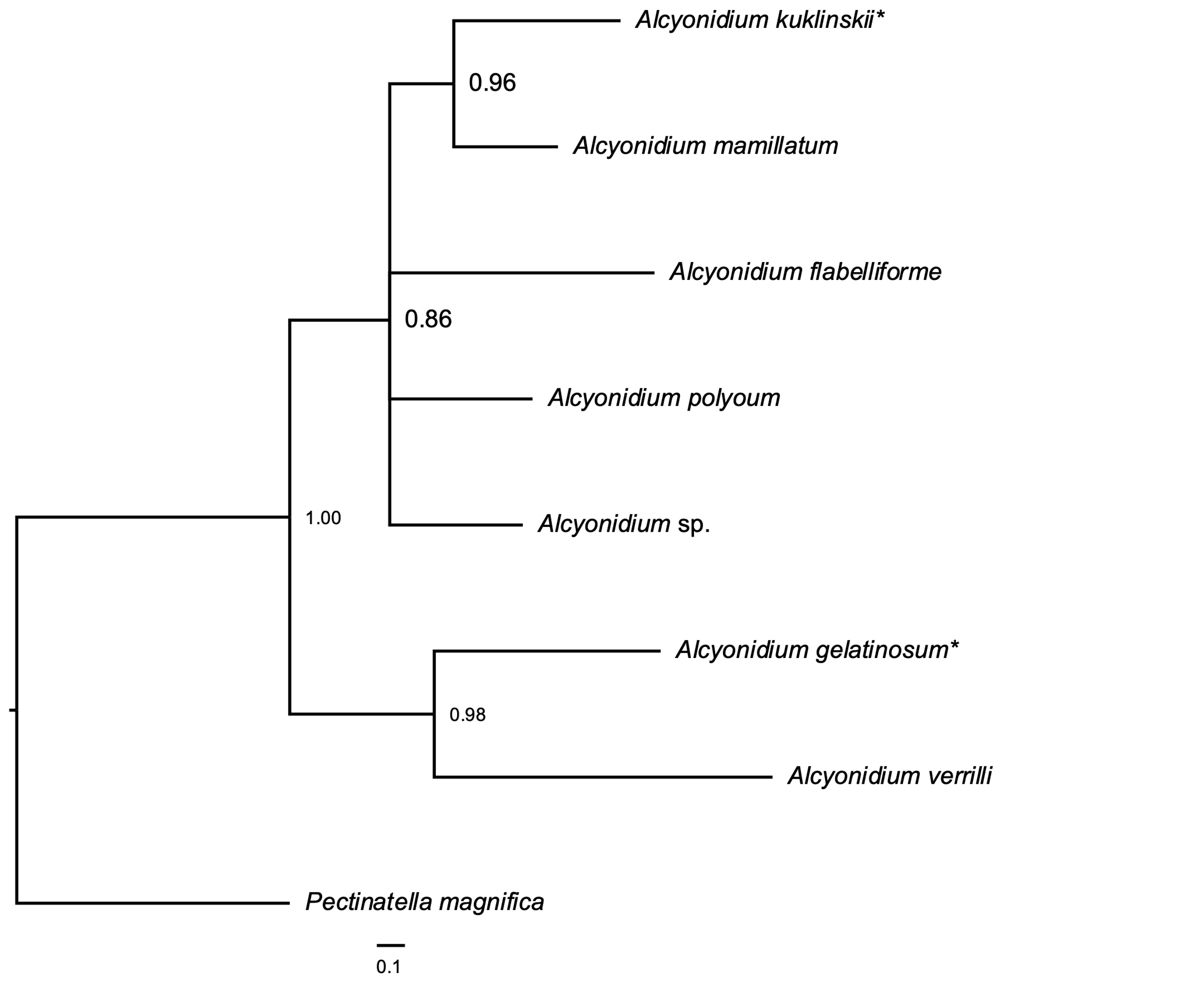

Supplement: Supplementary file 1 — Supplementary file1: Bayesian phylogenetic tree of Alcyonidium based on 646 unambiguously aligned nucleotide sites of the COI gene. Values on nodes represent posterior probabilities (based on last 75% of trees). Support values < 50% are not shown. The scale bar represents one substitutional change per 100 nucleotide positions. * Sequence was generated during this study (PNG 243 KB) [file 13127_2023_629_MOESM1_ESM.png]
